# Influence of believed AI involvement on the perception of digital medical advice

**DOI:** 10.1038/s41591-024-03180-7

**Published:** 2024-07-25

**Authors:** Moritz Reis, Florian Reis, Wilfried Kunde

**Affiliations:** 1 Institute of Psychology, Julius-Maximilians-Universität Würzburg, Würzburg, Germany; 2 Judge Business School, University of Cambridge, Cambridge, UK; 3 Medical Affairs, Pfizer Pharma GmbH, Berlin, Germany

**Keywords:** Health services, Psychology

## Abstract

Large language models offer novel opportunities to seek digital medical advice. While previous research primarily addressed the performance of such artificial intelligence (AI)-based tools, public perception of these advancements received little attention. In two preregistered studies (*n* = 2,280), we presented participants with scenarios of patients obtaining medical advice. All participants received identical information, but we manipulated the putative source of this advice (‘AI’, ‘human physician’, ‘human + AI’). ‘AI’- and ‘human + AI’-labeled advice was evaluated as significantly less reliable and less empathetic compared with ‘human’-labeled advice. Moreover, participants indicated lower willingness to follow the advice when AI was believed to be involved in advice generation. Our findings point toward an anti-AI bias when receiving digital medical advice, even when AI is supposedly supervised by physicians. Given the tremendous potential of AI for medicine, elucidating ways to counteract this bias should be an important objective of future research.

## Abstract (web summary)

In two preregistered studies involving 2,280 participants using simulated clinical scenarios, advice labeled as involving AI was evaluated as significantly less reliable and less empathetic compared with ‘human’-labeled advice.

## Main

Artificial intelligence (AI) holds enormous potential for the medical domain, for instance in analyzing medical images^1^ or detecting drug interactions^2^. Recent developments in the field of AI-based large language models (LLMs) have now given rise to numerous additional applications in healthcare. One use case that is becoming increasingly relevant from a public perspective is the use of LLMs when seeking medical advice^3^. To this end, popular LLM applications such as OpenAI’s ChatGPT offer low-threshold access to medical information, seemingly without the need to consult specialist literature or professional physicians. While there are considerable concerns regarding the use of LLMs in healthcare^4^, earlier research indicates that ChatGPT 4.0 already achieves levels of diagnostic accuracy on a par with human physicians^5^. In another study, physicians (unaware of who created the advice) even rated LLM-generated responses to medical queries as superior in quality and more empathetic than answers generated by human physicians^6^.

AI-generated medical advice, thus, is perceived as high quality as long as the AI authorship is undisclosed. Yet, research from various domains indicates reservations against such content as soon as the AI authorship becomes apparent (algorithm aversion^7^). While similar effects have been observed for the use of AI-based tools in the medical field^8^, little is known about the perception of novel LLM applications. Moreover, previous research in this field often relied on relatively small samples, lacked an experimental study design (for example, refs. ^9,10^) or focused solely on the physician’s perspective^11^. However, from the public’s point of view, not only the objective level of competence but also the subjective perception of the treating physician has a substantial influence on health-promoting behavior, treatment satisfaction and treatment outcome^12^. Similarly, when seeking medical advice in digital settings, not only technical performance but also the public’s perception of a new tool might prove decisive for its further dissemination and acceptance.

Within our work, we thus aim to explore the public’s perspective on LLM-generated medical advice in a controlled, experimental setting. Therefore, we conducted two preregistered experiments with large samples (study 1: *n* = 1,050 across various nationalities; study 2: *n* = 1,230, representative of the UK population in terms of age, gender and ethnicity). Within both studies, we investigated how labeling identical medical advice as generated either by a human physician or by an AI-supported chatbot affects how this information is perceived in terms of reliability, comprehensibility and empathy. As it is expected that AI will not replace but rather support human competencies in the future^13^, we further extend previous research by including a third group in which the information was labeled as generated by a human physician in collaboration with AI. In study 2, we additionally measured the individual willingness to follow the provided advice. Moreover, we assessed participants’ interest in testing the tool, which supposedly generated the previously encountered medical information by offering the opportunity to save a (fictious) link to a corresponding platform. Thus, our research abstracts from potential differences in the quality of AI- versus human-generated medical information. Instead, we focus on illuminating potential biases toward novel LLM-based tools as sources of medical advice from the public’s perspective.

**Fig. 1:**
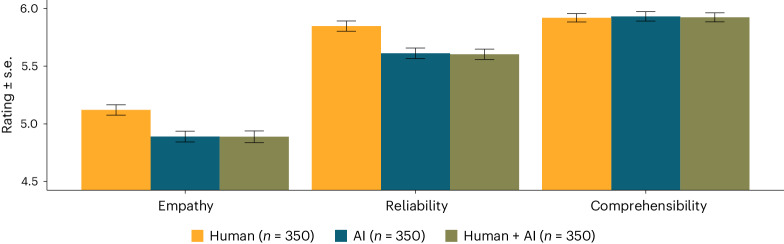
Average ratings for each dimension (empathy, reliability, comprehensibility) and author label (human, AI, human + AI). Samples for each author label condition were independent of each other, but each participant provided ratings for all dimensions. Ratings are aggregated across all four scenarios, and responses are scaled from 1 to 7, with higher values indicating stronger expressions of the respective dimension. Error bars show standard errors of the individual means.

**Fig. 2:**
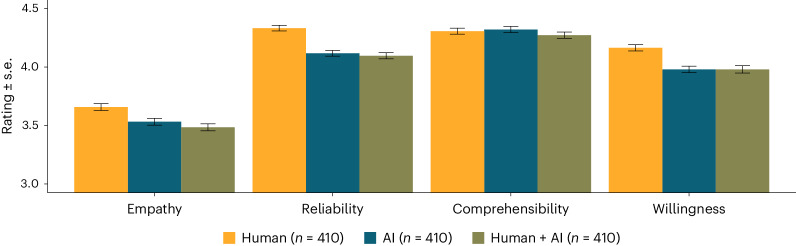
Average ratings for each dimension (empathy, reliability, comprehensibility, willingness to follow the advice) and author label (human, AI, human + AI). Samples for each author label condition were independent of each other, but each participant provided ratings for all dimensions. Ratings are aggregated across all four scenarios, and responses are scaled from 1 to 5, with higher values indicating stronger expressions of the respective dimension. Error bars show standard errors of the individual means.

Figure 1 shows average ratings for each dimension (empathy, reliability, comprehensibility) and author label (‘human’, ‘AI’, ‘human + AI’) in study 1. There was a significant main effect of the author label on empathy ratings, test statistics of corresponding one-way analysis of variance *F*(2, 1,047) = 7.98, *P* < 0.001, partial eta squared *η*_p_^2^ = 0.02. That is, the ‘human’ advice was perceived as significantly more empathic than ‘AI’ advice, test statistics of corresponding two-sample *t*-test *t*(698) = 3.58, *P* < 0.001, Cohen’s *d* = 0.27 95% confidence interval (CI) (0.12, 0.42), and ‘human + AI’ advice, *t*(698) = 3.44, *P* = 0.001, *d* = 0.26 95% CI (0.11, 0.41). There was no difference of empathy ratings between the ‘AI’ and ‘human + AI’ condition, *t* < 1. Reliability ratings differed significantly between different author labels, *F*(2, 1,047) = 9.68, *P* < 0.001, *η*_p_^2^ = 0.02. ‘Human’ advice was rated as significantly more reliable than ‘AI’ advice, *t*(698) = 3.72, *P* < 0.001, *d* = 0.28 95% CI (0.13, 0.43), and ‘human + AI’ advice, *t*(698) = 3.90, *P* < 0.001, *d* = 0.29 95% CI (0.15, 0.44). Reliability ratings did not differ between ‘AI’ and ‘human + AI’ advice, *t* < 1. Comprehensibility ratings were not affected by the author label, *F* < 1. Corresponding mixed-effect regression analyses are reported in Supplementary Information. Figure 2 shows the main results of study 2. For all analyses, the pattern mirrors the results observed in study 1. Thus, ‘human’ advice was evaluated as more empathic and more reliable, *t*s ≥ 3.01, *P*s ≤ 0.003, *d*s ≥ 0.21, but not as more comprehensible, *t*s < 1, compared with ‘AI’ and ‘human + AI’ advice. Along the same lines, participants indicated a significantly lower willingness to follow the provided advice when AI was believed to be involved in advice generation, *t*s ≥ 4.46, *P*s ≤ 0.001, *d*s ≥ 0.31. However, the share of participants who saved the link to the (fictious) platform (‘human’: 19.3%; ‘AI’: 18.5%; ‘human + AI’: 22.9%) did not differ between the ‘human’ and the ‘AI’ condition, non-standardized regression coefficient of logistic regression *b* = 0.05, test statistics of corresponding Wald test *z* = 0.27, *P* = 0.789, nor between the ‘human’ and the ‘human + AI’ condition, *b* = 0.22, *z* = 1.28, *P* = 0.200. Detailed results and explorative analyses on interindividual differences are provided in Supplementary Information.

Our findings may be based on a stronger association of the ‘human physician’ label with a mutual demonstration of care and respect, which are vital factors for successful patient–physician interactions^14^. Hence, our results reinforce that the public perceives physicians as more appropriate sources of medical information than AI-based tools. This outcome is in line with earlier findings on algorithm aversion^7^, particularly within the medical domain (for example, ref. ^11^). Conversely, the use of AI may have been perceived as ‘dehumanizing’^15^, a sentiment highlighted by the lower empathy scores for AI-labeled advice in both of our studies. A further explanation for the observed resistance to AI-generated medical advice might be the phenomenon of ‘uniqueness neglect’, wherein users believe AI may not adequately consider their individual characteristics. Consequently, explaining that even AI-generated advice processes and considers personal information provided by the individuals themselves could potentially increase acceptance^16^.

Our observation that human-labeled medical advice was not perceived as significantly more comprehensible than AI-labeled information indicates that the label effect exerted less influence on this dimension compared with factors such as reliability and empathy. Possibly, this dimension was perceived as more technical in nature and therefore less critical for sensitive medical settings. In this respect, AI could therefore perform on an equivalent level with human physicians. This observation is noteworthy as it indicates that AI was not generally evaluated more ‘negatively’, which can occur when judgment of one feature influences judgments of other features of the same evaluation object, known as halo effect^17^.

Furthermore, there was no effect of the author label on the decision to save a link to the platform where the provided responses supposedly were generated. Even though this finding does not necessarily imply that people would also follow AI-generated medical advice, it seems that members of the public at least show interest in corresponding AI-based tools. Whether this initial exploration would also lead to a long-term and unbiased use, however, is yet to be explored.

Both of our studies have limitations. First, to ensure high internal validity, participants in the experiments had to adopt the perspective of other individuals and therefore were unable to formulate their own inquiries. Moreover, the examined dialogs consisted of only one incoming question and a single subsequent response. Thus, our chosen setting was representative of brief interactions occurring on a digital medical platform, while not capturing extensive interactions as those in face-to-face doctor–patient consultations. We consider such a more interactive but also less controlled environment as an intriguing approach for future research.

Our findings indicate a bias against medical advice labeled as AI-generated, regardless of additional supervision by human physicians. Along the same lines, previous research has shown that the public’s reservations toward medical AI persist even when AI-generated content is medically supervised^18^. Considering the expected surge of AI in healthcare and the immense potential for human–AI collaboration, this finding raises notable concerns. To address this bias, in addition to the general public, other stakeholders, such as physicians and insurance providers, will need to be engaged accordingly. Interestingly, another study showed that if people were assured that humans would remain unequivocally in the decision-making position, the combination of human and AI achieved significantly higher levels of trust than without this assurance^19^. Consequently, the specific framing of the involvement of AI in generating and delivering medical advice may be pivotal for its public acceptance.

## Methods

### Ethics and inclusion

All participants received detailed instructions regarding their task, provided informed consent and were debriefed about the study purpose at the end of the experiment. Both of our studies were conducted in accordance with the Declaration of Helsinki. We received formal approval from the ethics committee of the Institute of Psychology of the Faculty of Human Sciences of the University of Würzburg before conducting the studies (GZEK 2023-66).

### Study 1

#### Participants

The study was programmed with lab.js (version 20.2.4 (ref. ^20^)) and hosted on a private web server. We recruited 1,090 participants via Prolific (www.prolific.com), among which 3.7% (*n* = 40) did not finish the experiment and were thus excluded from the analysis (final sample size: 1,050; 350 per author label group; self-reported gender identity: 555 males, 489 females, 5 non-binaries, 1 prefer not to say; age: *M* = 33.0 years, s.d. = 11.5 years). This sample size provided high statistical power to detect even small effects of the author label on reported ratings (1 − *β* = 95% for *d* ≥ 0.273, *α* = 0.05 (where *β* and *α* are the type II and type I error probabilities, respectively), two-sample *t*-test, two-tailed testing, computed in R, version 4.1.1, via the power.t.test function of the stats package version 3.6.2). The majority of this sample indicated a university degree as their highest level of education (3 no formal qualification, 53 secondary education, 265 high school, 500 bachelor, 195 master, 28 PhD, 6 prefer not to say). Participants reported about 60 different nationalities, with South Africa (*n* = 262), the United Kingdom (*n* = 174) and Poland (*n* = 76) mentioned most frequently.

#### Materials

##### Case reports

The case reports used in this study address four distinct medical topics: smoking cessation, colonoscopy, agoraphobia and reflux disease (Supplementary Figs. 1–4). Each of these scenarios comprises a brief dialog consisting of an inquiry as it might be presented by a medical layperson using a chat interface on a digital health platform, along with an appropriate response to this inquiry. The queries were constructed and validated by a certified physician. To generate the responses in a style similar to that of popular LLMs, the preceding inquiries were used as prompts for OpenAI’s ChatGPT 3.5. The resultant outcomes were edited in their formulations, supplemented with additional information and scrutinized for medical accuracy by a certified physician. Thus, all case reports constituted a collaboration between AI and a human physician, regardless of the information provided to the participants during the experiment.

##### Scales

Participants evaluated the presented case reports regarding perceived reliability, comprehensibility and empathy. By using these categories, we closely adhered to existing literature on key evaluation criteria from the patient’s perspective in doctor–patient interactions (see refs. ^6,21^ for ‘reliability’ and ‘empathy’ and ref. ^22^ for ‘comprehensibility’). Moreover, these three dimensions allowed us to cover different facets of medical dialogs in a reasonably comprehensive and distinct manner. With ‘reliability’, we addressed the assessment of the content of the medical advice (content-related component). With ‘comprehensibility’, we recorded the public understandability and how accessible the information was structured (format-related component). Finally, with ‘empathy’, we captured the transfer of information on an emotional interpersonal level (interaction-related component). As no established survey instruments with practice-proven suitability for the present research question exist, we developed novel scales closely aligned with best practices in this field. That is, we decided on a relatively low number of response options with individual, unambiguous labels and used symmetrical scales with nonoverlapping categories^23,24^. The final 7-point Likert scales went from ‘extremely unreliable’ to ‘extremely reliable’, from ‘extremely difficult to understand’ to ‘extremely easy to understand’ and from ‘extremely unempathic’ to ‘extremely empathic’.

For the ‘AI’-label group, ratings for each scale were positively correlated with participants’ attitudes toward AI (perceived opportunities compared with risks, perceived impact for healthcare), *P*s ≤ 0.022, thus pointing to high conceptual validity of our scales.

#### Experimental design and procedure

We used a unifactorial between-subject design, with the manipulated factor being the supposed author of the presented medical information (human, AI, human + AI; Supplementary Fig. 5). Participants were instructed to carefully read all scenarios that were presented in random order. Afterward, we assessed participants’ attitudes toward AI. Hence, we inquired about their frequency of using AI-based tools (response options: never, rarely, occasionally, frequently, very frequently), their perception of the impact of AI on healthcare (response options: no, minor, moderate, significant, highly significant) and whether they view the integration of AI in healthcare as presenting more risks or opportunities (response options: more risks, neutral, more opportunities). Finally, we collected demographic information on gender, age, educational level and nationality.

#### Data treatment and analyses

We preregistered our analysis plan, data collection strategy and the experimental design (https://osf.io/6trux).

Data analysis was conducted in R version 4.1.1 (R Core Team). A separate analysis of variance was calculated for each rating dimension (reliability, comprehensibility, empathy), using the supposed author of the medical advice as a between-subject factor (human, AI, human + AI). Significant main effects were followed by two-sample *t*-tests (two-tailed), comparing all factor levels. Cohen’s *d* is reported as a measure of effect size, which is calculated with the t_out function of the schoRsch package version 1.10 in R (ref. ^25^). To account for multiple testing, we used the Holm–Bonferroni method to adjust the significance level (*α*).

As an additional analysis, which we did not preregister, a separate mixed-effect regression analysis was calculated for each rating dimension (reliability, comprehensibility, empathy), using the supposed author of the medical advice (human, AI, human + AI) as a fixed factor and the different scenarios as well as the individual participant as random factors (intercepts). The author label condition was dummy coded with the ‘human’ condition as the reference category. We report absolute values for all statistics and *P* values were calculated using Satterthwaite’s method. Corresponding results are reported in Supplementary Information.

### Study 2

#### Participants

For study 2, we recruited a new sample of 1,456 participants via Prolific, among which 6.1% (*n* = 89) did not finish the experiment and were thus excluded from the analysis. As preregistered, we further excluded datasets of participants who failed the attention check (that is, indicated the wrong author label at the end of the study; see ‘Materials and procedure’ for details). This applied to 9.4% (*n* = 137) of our participants. Thus, our final sample consisted of 1,230 individuals (410 per author label group). For our second study, we exclusively recruited participants from the United Kingdom and our sample was representative of the UK population in terms of age, gender and ethnicity (self-reported gender identity: 595 males, 619 females, 10 non-binaries, 6 prefer not to say; age: *M* = 47.3 years, s.d. = 15.6 years). Our sample size provided high statistical power to detect even small effects of the author label on reported ratings (1 − *β* = 90% for *d* ≥ 0.270, *α* = 0.01, two-sample *t*-test, two-tailed testing, computed in R, version 4.1.1, via the power.t.test function of the statistics package). The majority of this sample indicated a university degree as their highest level of education (12 no formal qualification, 146 secondary education, 325 high school, 532 bachelor, 167 master, 40 PhD, 8 prefer not to say).

#### Materials and procedure

Within our second experiment, we used the same case reports as for study 1. Again, we used a unifactorial between-subject design, with the manipulated factor being the supposed author of the presented medical information (human, AI, human + AI; Supplementary Fig. 5). However, in contrast to study 1, the author label was manipulated only via text instead of via additional symbols. The experimental procedure was similar to that of study 1, but we used two additional measures of preference. Thus, in addition to perceived reliability, comprehensibility and empathy, we also measured the individual willingness to follow the provided advice. To further test the robustness of our survey instruments, we also slightly adapted the scales on which participants rated the respective dimensions. That is, we used 5-point Likert scales (instead of the 7-point scales used in study 1), going from ‘very unreliable’ to ‘very reliable’, from ‘very difficult to understand’ to ‘very easy to understand’, from ‘very unempathic’ to ‘very empathic’ and from ‘very unwilling’ to ‘very willing’. Moreover, at the end of the experiment, participants had the opportunity to save a (fictious) link to the platform and tool, which supposedly generated the previously encountered responses. This tool was framed depending on the experimental condition (‘The previous scenarios where exemplary conversations from a digital platform where users can engage in conversations with a licensed medical doctor (an AI-supported chatbot) regarding medical queries. (All responses on this platform are reviewed by a licensed medical doctor and may be supplemented or revised if necessary.)’). Participants could save this link by clicking on a corresponding button. For each rating dimension, there was a positive relation with the decision to save the link, *P*s ≤ 0.012. Moreover, similar to study 1, for the AI condition, attitudes toward AI (perceived opportunities and impact) were positively correlated with ratings in each domain, *P*s ≤ 0.001, thus again supporting the validity of our scales. At the end of the study, we again queried participants’ attitudes toward AI and demographic information. In addition, we also assessed participants’ patient status (‘Based on your current health status, would you describe yourself as a patient?’; response options: yes, no, prefer not to say) and whether they work in a healthcare-related profession or received a healthcare-related training (‘Based on your training or current profession, would you describe yourself as a healthcare professional?’; response options: yes, no, prefer not to say). If the latter question was answered with ‘yes’, participants could also indicate their exact profession. Finally, as an attention check, we asked participants who the stated source of the provided medical responses was (‘a licensed medical doctor’, ‘an AI-supported chatbot’, ‘an AI-supported chatbot, revised and supplemented by a licensed medical doctor’).

#### Data treatment and analyses

We preregistered our analysis plan, data collection strategy and the experimental design (https://osf.io/wn6mj).

Again, data analysis was conducted in R version 4.1.1 (R Core Team). For each rating dimension (reliability, comprehensibility, empathy, willingness to follow), a similar mixed-effect regression analysis was calculated as for study 1. Significant treatment effects were followed by two-sample *t*-tests (two-tailed), comparing all factor levels. Similar to study 1, Cohen’s *d* is reported as a measure of effect size. Furthermore, we calculated a binomial logistic regression of the decision to press the ‘save link’ button (yes or no), using the author label condition (human, AI, human + AI) as a fixed factor and the individual participant as a random factor (intercept). The author label condition was dummy coded with the ‘human’ condition as the reference category. We report absolute values for all statistics and *P* values were calculated using Satterthwaite’s method. Again, the Holm–Bonferroni method was applied to account for multiple testing.

As an exploratory analysis, we correlated individual attitudes toward AI (usage frequency, perceived risk, perceived impact) and further individual characteristics (age, gender, level of education, patient status, healthcare-related profession or training) with ratings of reliability, comprehensibility, empathy, willingness to follow and the decision to save the link to the fictious platform. These calculations were conducted separately for the ‘AI’ and the ‘human + AI’ group. Results for all exploratory analyses are reported in Supplementary Information.

### Reporting summary

Further information on research design is available in the Nature Portfolio Reporting Summary linked to this article.

## Online content

Any methods, additional references, Nature Portfolio reporting summaries, source data, extended data, supplementary information, acknowledgements, peer review information; details of author contributions and competing interests; and statements of data and code availability are available at 10.1038/s41591-024-03180-7.

## Supplementary information

Supplementary Information Supplementary Figs. 1–5 and Results.

Reporting Summary

## Data Availability

Underlying data for both studies can be found via OSF at https://osf.io/cxb7s/.

## Code availability

Analysis code for both studies can be found via OSF at https://osf.io/cxb7s/.
