# Supplementary material for: Influence of believed AI involvement on the perception of digital medical advice
Source: Nat Med. 2024 Jul 25;30(11):3098–100. doi: 10.1038/s41591-024-03180-7 (PMC11564086; doi:10.1038/s41591-024-03180-7)
Supplement: Supplementary file 1 — Supplementary Figs. 1–5 and Results. [file 41591_2024_3180_MOESM1_ESM.pdf]

nature medicine

Supplementary information https://doi.org/10.1038/s41591-024-03180-7

Influence of believed AI involvement on the perception of digital medical advice

In the format provided by the authors and unedited

Supplementary Information

Fig. S1-S4: Medical scenarios

Fig. S5: Author labels

Study 1: Mixed-effect regression analysis

Study 2: Detailed results, mixed-effect regression analysis and interindividual differences (Tables S1-S4)

Question: Hello, I've been experiencing frequent heartburn, a burning sensation in my chest, occasional regurgitation of sour fluids into my throat, and difficulty swallowing. I'm wondering about the possible causes of these symptoms and whether it's necessary to see a doctor. What options do I have to get it under control?

<<< Author label >>>

Response: Hello, thank you for sharing your concerns.

The symptoms you've described, such as frequent heartburn, chest burning, regurgitation of sour fluids, and difficulty swallowing, could align with Gastroesophageal Reflux Disease (GERD). GERD occurs when stomach acid regularly moves back into the tube linking the mouth and stomach (esophagus), causing irritation to its lining. Many people experience acid reflux from time to time. However, since these are serious symptoms and there are always various underlying conditions to be considered, it's advisable to seek medical evaluation to confirm the diagnosis.

Treatment options for GERD often include lifestyle changes like dietary modifications, elevating the head during sleep, and avoiding trigger foods or beverages. Medications, such as proton pump inhibitors or antacids, can also help manage symptoms. In severe cases, surgery might be considered.

Given your symptoms and their impact on daily life, I highly recommend consulting a healthcare provider for a proper diagnosis and personalized treatment plan. If you need further clarification or have additional questions, feel free to ask.

Fig S1. Scenario on reflux disease.

Question: Hi there, I've heard about getting a colonoscopy at my age and I'm curious if it's a good idea for me. Can you tell me what diseases or problems a colonoscopy can find? Also, are there any risks I should know about? I just want to know if it's a good thing for someone like me to do. Any help would be appreciated. Thanks!

<<< Author label >>>

Response: Hello, thank you for reaching out.

A routine colonoscopy is a procedure used to check for changes in the large intestine (colon) and rectum. It's generally recommended for individuals in middle age and older to investigate intestinal signs and detect early signs of colorectal cancer or other digestive issues. During a colonoscopy, we can also identify conditions like polyps, which are small growths that can sometimes turn into cancer. Additionally, it helps us detect any inflammation, bleeding, or signs of conditions like Crohn's disease or ulcerative colitis.

During the procedure, after receiving sedation, you'll lie on your side as a colonoscope is inserted into your rectum, allowing the doctor to examine your colon's lining using a tiny camera and pump air or carbon dioxide for better visibility. This process might cause cramping or a feeling of needing to pass stool. The doctor can also perform procedures like taking biopsies or removing abnormal tissue, and the entire procedure usually lasts 30 to 60 minutes.

As for risks, while colonoscopies are generally safe, potential complications might involve reactions to sedation, bleeding from tissue sample removal (biopsy), or in rare cases, a tear in the colon or rectum wall (perforation). Before deciding, it's important to discuss with your healthcare provider to assess if it's the right choice for you based on your age, your medical history and any current symptoms you might have. Feel free to ask if you need more information on this procedure or want to discuss further.

Fig S2. Scenario on colonoscopy.

Question: Hi, I'm a long-term smoker and I'm looking for information on how to quit smoking. Are there any methods available to help me smoke less or quit altogether? Could you please explain how these methods work and what their success rates are? I'm really motivated to quit and would appreciate any guidance or advice you can offer. Thank you!

<<< Author label >>>

Response: Hello, it’s great to hear that you are motivated to quit smoking.

In fact, there are several methods available to help reduce or quit smoking, such as nicotine replacement therapies (like patches, gums, or lozenges), prescription medications, and behavioral therapies. Nicotine replacement therapies work by providing controlled doses of nicotine to curb cravings, while prescription medications target receptors in the brain to reduce the urge to smoke. Behavioral therapies involve counseling or support groups, offering coping strategies to manage triggers and habits associated with smoking.

Success rates can vary based on individual factors. Only about five percent of former smokers, depending on the study, remain smoke-free after a year if they quit without any assistance, solely relying on their own willpower. With appropriate help, the rate increases to 40 to 50 percent. E-cigarettes, while gaining popularity as cigarette alternatives, haven't shown superior safety or effectiveness compared to nicotine-replacement medications for smoking cessation.

Ultimately, success in quitting smoking can be enhanced by finding the method or combination of methods that suit you best and seeking continuous support from healthcare providers or support groups. If you need further information, feel free to ask.

Fig S3. Scenario on smoking cessation.

Question: Hello there, I've been experiencing certain symptoms that are making it challenging for me to leave my home or be in crowded places. I often feel intense fear, sweating, rapid heartbeat, and dizziness when I consider going out. These feelings have been affecting my daily life, and I'm unsure if it's a specific health condition. What might be causing these symptoms and are there any potential treatments available? Thank you in advance for your support

<<< Author label >>>

Response: Hello, thank you for taking this step and seeking assistance now.

The symptoms you've described align with characteristics of agoraphobia, a type of anxiety disorder. Agoraphobia can make it challenging to navigate public spaces due to fear of panic attacks or feelings of being trapped. The anxiety is caused by fear that there's no easy way to escape if the anxiety gets overwhelming. Various factors contribute to the development of agoraphobia, involving genetics, health conditions, personality, stress und learning experiences.

Treatment for agoraphobia typically combines psychotherapy, like cognitive behavioral therapy (CBT), and medication, offering effective strategies to alleviate symptoms and enhance recovery. CBT focuses on developing coping skills to manage anxiety, directly challenging worries, and gradually re-engaging in avoided activities. Exposure therapy, a crucial part of treatment, involves gradual, controlled exposure to feared situations. Support from a trusted person during appointments could offer comfort and guidance if needed.

Medication, primarily antidepressants like selective serotonin reuptake inhibitors (SSRIs) are commonly prescribed for treating agoraphobia. These antidepressants are more effective than anti-anxiety medications for managing agoraphobia symptoms. Benzodiazepines, a type of anti-anxiety medicine, may be used briefly to reduce acute anxiety.

It's essential to seek professional evaluation to confirm the diagnosis and discuss appropriate treatment options. Given the impact of these symptoms on your daily life, I strongly recommend consulting a healthcare provider or mental health professional for a proper evaluation. Seeking professional help with these symptoms may not be easy. If I can assist you in any way, I'd be happy to!

Fig S4. Scenario on agoraphobia.

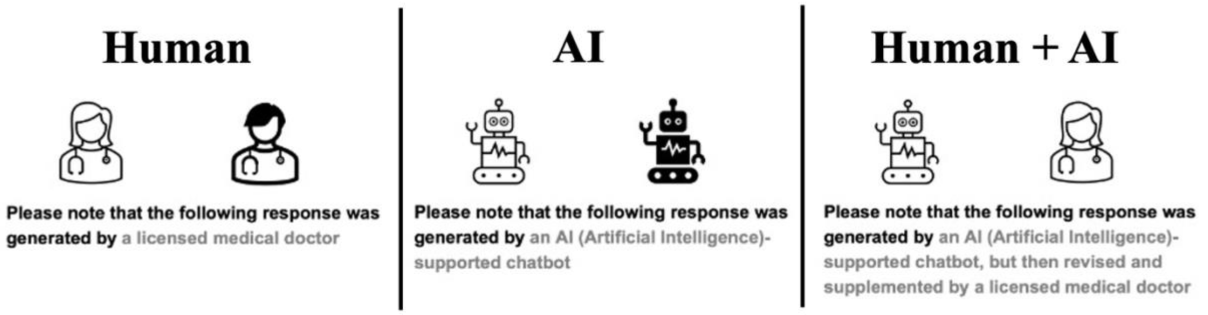

Fig S5. Author labels for each condition in study 1 (Human, AI, Human + AI). For each participant in the "Human + AI" condition we determined randomly whether the symbol for a male or female doctor was presented. In study 2, only text (no symbols) was used to manipulate the author label.

Study 1

Mixed-effect regression analysis

Empathy ratings were significantly higher for “Human” advice compared to “AI”, b = 0.23, $t(1047.00) = 3.45$, $p < .001$, and “Human + AI” advice, b = 0.23, $t(1047.00) = 3.47$, $p < .001$. Along the same lines, “Human” advice was rated as significantly more reliable than AI, b = 0.24, $t(1047.00) = 3.73$, $p < .001$, and “Human + AI” advice, b = 0.25, $t(1047.00) = 3.88$, $p < .001$. However, perceived comprehensibility did neither differ significantly between “Human” and “AI” advice, b = 0.01, $t < 1$, nor between “Human” and “Human + AI” advice, $b < 0.01$, $t < 1$.

Study 2

Detailed results

"Human" advice received significantly higher empathy ratings than "AI" advice, $t(818) = 3.01$, $p = .003$, $d = 0.21$ 95%-CI [0.07, 0.35], and "Human + AI" advice, $t(818) = 4.12$, $p < .001$, $d = 0.29$ 95%-CI [0.15, 0.43]. No such difference emerged between "AI" and "Human + AI" advice, $t(818) = 1.14$, $p = .253$, $d = 0.08$ 95%-CI [-0.06, 0.22]. "Human" advice was rated as significantly more reliable than "AI" advice, $t(818) = 6.23$, $p < .001$, $d = 0.44$ 95%-CI [0.30, 0.57], and "Human + AI" advice, $t(818) = 6.60$, $p < .001$, $d = 0.46$ 95%-CI [0.32, 0.60]. There was no difference between "AI" and "Human + AI" advice for reliability, $t < 1$. Participants indicated a significantly higher willingness to follow "Human" advice than "AI" advice, $t(818) = 4.85$, $p < .001$, $d = 0.34$ 95%-CI [0.20, 0.48] or "Human + AI" advice, $t(818) = 4.46$, $p < .001$, $d = 0.31$ 95%-CI [0.17, 0.45]. There was no difference between "AI" and "Human + AI" advice, $t < 1$.

Mixed-effect regression analyses

Empathy ratings were significantly higher for "Human" advice compared to "AI", $b = 0.13$, $t(1227.00) = 2.98$, $p = .003$, and "Human + AI" advice, $b = 0.17$, $t(1227.00) = 4.13$, $p < .001$. Along the same lines, "Human" advice was rated as significantly more reliable than "AI" advice, $b = 0.22$, $t(1227.00) = 6.06$, $p < .001$, and "Human + AI" advice, $b = 0.24$, $t(1227.00) = 6.64$, $p < .001$. However, perceived comprehensibility did neither differ significantly between "Human" and "AI" advice, $b = 0.01$, $t < 1$, nor between "Human" and "Human + AI" advice, $b = 0.03$, $t < 1$. Finally, the willingness to follow "Human" advice was significantly higher compared to "AI" advice, $b = 0.18$, $t(1227.00) = 4.58$, $p < .001$, and "Human + AI" advice, $b = 0.18$, $t(1227.00) = 4.58$, $p < .001$.

Interindividual differences

Tables S1-S4 present the correlations of individual characteristics (attitudes toward AI, demographic information, patient status, healthcare-related profession or training) with the evaluations of the medical advice and the decision to save the link to the fictious tool.

Table S1. Correlations of behavior in study 2 (ratings of empathy, reliability, comprehensibility and willingness as well as the decision to save the link) and attitudes toward AI (use, impact, risk) for the "AI" condition. Test statistics are based on Pearsons's product moment correlation coefficient (two-sided testing, no adjustments for multiple comparisons were made). Higher values for "AI risk" indicate that AI is rather seen as an opportunity than a risk for healthcare. *** $p < 0.001$, ** $p < 0.01$, * $p < 0.05$.

Variables	*r*	*t*	*df*	*p*
Empathy * AI use	.03	0.69	408	.489
Empathy * AI impact	.26***	5.37	408	< .001
Empathy * AI risk	.25***	5.28	408	< .001
Reliability * AI use	.03	0.51	408	.611
Reliability * AI impact	.25***	5.28	408	< .001
Reliability * AI risk	.24***	4.94	408	< .001
Comprehensibility * AI use	.02	0.32	408	.746
Comprehensibility * AI impact	.30***	6.32	408	< .001
Comprehensibility * AI risk	.22***	4.66	408	< .001
Willingness * AI use	< |.01|	0.02	408	.988
Willingness * AI impact	.32***	6.88	408	< .001
Willingness * AI risk	.27***	5.62	408	< .001
Link * AI use	.27***	5.72	408	< .001
Link * AI impact	.02	0.43	408	.664
Link * AI risk	.14**	2.76	408	.006

Table S2. Correlations of behavior in study 2 (ratings of empathy, reliability, comprehensibility and willingness as well as the decision to save the link) and attitudes toward AI (use, impact, risk) for the "Human + AI" condition. Test statistics are based on Pearsons's product moment correlation coefficient (two-sided testing, no adjustments for multiple comparisons were made). Higher values for "AI risk" indicate that AI is rather seen as an opportunity than a risk for healthcare. *** $p < 0.001$, ** $p < 0.01$, * $p < 0.05$.

Variables	*r*	*t*	*df*	*p*
Empathy * AI use	.07	1.50	408	.135
Empathy * AI impact	.20***	4.18	408	< .001
Empathy * AI risk	.19***	3.98	408	< .001
Reliability * AI use	.03	0.67	408	.502
Reliability * AI impact	.30***	6.44	408	< .001
Reliability * AI risk	.24***	5.10	408	< .001
Comprehensibility * AI use	- .01	0.16	408	.873
Comprehensibility * AI impact	.18***	3.61	408	< .001
Comprehensibility * AI risk	.19***	3.95	408	< .001
Willingness * AI use	.01	0.26	408	.792
Willingness * AI impact	.29***	6.03	408	< .001
Willingness * AI risk	.38***	8.31	408	< .001
Link * AI use	.18***	3.69	408	< .001
Link * AI impact	.12*	2.53	408	.012
Link * AI risk	.18***	3.71	408	< .001

Table S3. Correlations of behavior in study 2 (ratings of empathy, reliability, comprehensibility and willingness as well as the decision to save the link) and individual characteristics (age, education, gender, patient status, healthcare-related profession or training) for the "AI" condition. Test statistics are based on Pearsons's product moment correlation coefficient (two-sided testing, no adjustments for multiple comparisons were made). Gender is coded with "male" = 0, "female" = 1; patient status is coded with "no patient" = 0, "patient" = 1; profession is coded with "no healthcare-related profession or training" = 0, "healthcare-related profession or training" = 1. *** $p < 0.001$, ** $p < 0.01$, * $p < 0.05$.

Variables	*r*	*t*	*df*	*p*
Empathy * Age	.19***	3.89	408	< .001
Empathy * Education	- .07	1.35	406[1]	.179
Empathy * Gender	.02	0.46	407[1]	.647
Empathy * Patient status	.15**	2.99	401[1]	.003
Empathy * Profession	- .04	0.71	405[1]	.481
Reliability * Age	.12*	2.53	408	.012
Reliability * Education	- .18***	3.60	406[1]	< .001
Reliability * Gender	.04	0.76	407[1]	.448
Reliability * Patient status	.05	1.06	401[1]	.292
Reliability * Profession	- .02	0.42	405[1]	.672
Comprehensibility * Age	.09	1.88	408	.061
Comprehensibility * Education	- .11*	2.22	406[1]	.027
Comprehensibility * Gender	.09	1.82	407[1]	.069
Comprehensibility * Patient status	.07	1.42	401[1]	.158
Comprehensibility * Profession	- .07	1.38	405[1]	.168
Willingness * Age	.19***	3.91	408	< .001
Willingness * Education	- .15**	3.06	406[1]	.002
Willingness * Gender	< .01	< 0.01	407[1]	.998
Willingness * Patient status	.10*	2.10	401[1]	.037
Willingness * Profession	- .02	0.48	405[1]	.631
Link * Age	- .06	1.12	408	.265
Link * Education	.10*	2.09	406[1]	.038
Link * Gender	- .06	1.12	407[1]	.265
Link * Patient status	.06	1.12	401[1]	.266
Link * Profession	.11*	2.21	405[1]	.028

[1] Reduced degrees of freedom, because only male and female participants (gender) or respectively only participants who did not chose the "prefer not to say" option (patient status, profession, education) were included in this analysis.

Table S4. Correlations of behavior in study 2 (ratings of empathy, reliability, comprehensibility and willingness as well as the decision to save the link) and individual characteristics (age, education, gender, patient status, healthcare-related profession or training) for the "Human + AI" condition. Test statistics are based on Pearsons's product moment correlation coefficient (two-sided testing, no adjustments for multiple comparisons were made). Gender is coded with "male" = 0, "female" = 1; patient status is coded with "no patient" = 0, "patient" = 1; profession is coded with "no healthcare-related profession or training" = 0, "healthcare-related profession or training" = 1. *** $p < 0.001$, ** $p < 0.01$, * $p < 0.05$.

Variables	*r*	*t*	*df*	*p*
Empathy * Age	.09	1.80	408	.073
Empathy * Education	.01	0.19	404[1]	.851
Empathy * Gender	.07	1.43	399[1]	.153
Empathy * Patient status	.07	1.32	400[1]	.187
Empathy * Profession	.09	1.76	403[1]	.080
Reliability * Age	.09	1.74	408	.083
Reliability * Education	- .02	0.39	404[1]	.697
Reliability * Gender	.09	1.85	399[1]	.065
Reliability * Patient status	.12*	2.34	400[1]	.020
Reliability * Profession	.04	0.79	403[1]	.430
Comprehensibility * Age	.07	1.50	408	.134
Comprehensibility * Education	- .02	0.31	404[1]	.754
Comprehensibility * Gender	.15**	3.12	399[1]	.002
Comprehensibility * Patient status	.10	1.92	400[1]	.056
Comprehensibility * Profession	.03	0.60	403[1]	.546
Willingness * Age	.10*	2.01	408	.045
Willingness * Education	.03	0.66	404[1]	.510
Willingness * Gender	.08	1.64	399[1]	.101
Willingness * Patient status	.11*	2.19	400[1]	.029
Willingness * Profession	.05	1.08	403[1]	.280
Link * Age	- .08	1.56	408	.121
Link * Education	.05	1.03	404[1]	.304
Link * Gender	- .04	0.86	399[1]	.390
Link * Patient status	.03	0.50	400[1]	.617
Link * Profession	.01	0.12	403[1]	.903

[1] Reduced degrees of freedom, because only male and female participants (gender) or respectively only participants who did not chose the "prefer not to say" option (patient status, profession, education) were included in this analysis.
