# Supplementary material for: Influence of believed AI involvement on the perception of digital medical advice
Source: Nat Med. 2024 Jul 25;30(11):3098–100. doi: 10.1038/s41591-024-03180-7 (PMC11564086; doi:10.1038/s41591-024-03180-7)
Supplement: Supplementary file 2 — Reporting Summary [file 41591_2024_3180_MOESM2_ESM.pdf]

nature portfolio

Corresponding author(s): Moritz Reis
Last updated by author(s): Jun 30, 2024

Reporting Summary

Nature Portfolio wishes to improve the reproducibility of the work that we publish. This form provides structure for consistency and transparency in reporting. For further information on Nature Portfolio policies, see our Editorial Policies and the Editorial Policy Checklist.

Statistics

For all statistical analyses, confirm that the following items are present in the figure legend, table legend, main text, or Methods section.

n/a	Confirmed	
☐	☒	The exact sample size (*n*) for each experimental group/condition, given as a discrete number and unit of measurement
☐	☒	A statement on whether measurements were taken from distinct samples or whether the same sample was measured repeatedly
☐	☒	The statistical test(s) used AND whether they are one- or two-sided<br>*Only common tests should be described solely by name; describe more complex techniques in the Methods section.*
☐	☒	A description of all covariates tested
☐	☒	A description of any assumptions or corrections, such as tests of normality and adjustment for multiple comparisons
☐	☒	A full description of the statistical parameters including central tendency (e.g. means) or other basic estimates (e.g. regression coefficient) AND variation (e.g. standard deviation) or associated estimates of uncertainty (e.g. confidence intervals)
☐	☒	For null hypothesis testing, the test statistic (e.g. *F*, *t*, *r*) with confidence intervals, effect sizes, degrees of freedom and *P* value noted<br>*Give P values as exact values whenever suitable.*
☒	☐	For Bayesian analysis, information on the choice of priors and Markov chain Monte Carlo settings
☒	☐	For hierarchical and complex designs, identification of the appropriate level for tests and full reporting of outcomes
☐	☒	Estimates of effect sizes (e.g. Cohen's *d*, Pearson's *r*), indicating how they were calculated

Our web collection on statistics for biologists contains articles on many of the points above.

Software and code

Policy information about availability of computer code

Data collection	The study was programmed with lab.js (version 20.2.4), hosted on a private web server and we recruited our sample via Prolific (www.prolific.com).
Data analysis	We analyzed our data with R (version 4.1.1). The analysis code can be found on the OSF (https://osf.io/cxb7s/). For our power analyses, we used the power.t.test function of the stats package (version 3.6.2) in R. Cohen's d as a measure of effect size was calculated with the t_out function of the schoRsch package (version 1.10) in R.

For manuscripts utilizing custom algorithms or software that are central to the research but not yet described in published literature, software must be made available to editors and reviewers. We strongly encourage code deposition in a community repository (e.g. GitHub). See the Nature Portfolio guidelines for submitting code & software for further information.

Data

Policy information about availability of data

All manuscripts must include a data availability statement. This statement should provide the following information, where applicable:

- Accession codes, unique identifiers, or web links for publicly available datasets
- A description of any restrictions on data availability
- For clinical datasets or third party data, please ensure that the statement adheres to our policy

The underlying data can be found on the OSF (https://osf.io/cxb7s/).

April 2023

Research involving human participants, their data, or biological material

Policy information about studies with human participants or human data. See also policy information about sex, gender (identity/presentation), and sexual orientation and race, ethnicity and racism.

Reporting on sex and gender	Participants indicated their gender identity via self-report (male, female, non-binary, prefer not to say). Self-reported gender identity for study 1: 555 males, 489 females, 5 non-binaries, 1 prefer not to say. Self-reported gender identity for study 2: 595 males, 619 females, 10 non-binaries, 6 prefer not to say. For study 1, we did not use this information for analyses. However, for study 2, we analyzed the relationship of self-reported gender identity and behavior in the experiment (see supplementary information for details).
Reporting on race, ethnicity, or other socially relevant groupings	We did not collect data on race or ethinicity but for study 1 we collected data on participants' nationality. Responses for nationality were entered via self-report in a free-text box but we did not use this information for analyses. For study 1, participants reported about 60 different nationalities, with South Africa (n = 262), the UK (n = 174) and Poland (n = 76) mentioned most frequently. For study 2, we exclusively recruited participants from the UK.
Population characteristics	See "Research sample" below.
Recruitment	Participants were recruited via Prolific (www.prolific.com). As the experiment was conducted in English, participants required sufficient language skills to participate. Moreover, due to the online setup, a corresponding device and Internet access was required. These requirements might had the effect that people with low socio-economic status were underrepresented in our samples. As people with low socio-economic status have been found to be relatively more aversive against technological innovations like AI, this could render our findings on biases against AI as rather conservative. Moreover, participants who are in general interested in scientific research and taking part in corresponding studies might be overrepresented in our samples.
Ethics oversight	This study was approved by the ethics committee of the Institute for Psychology of the faculty for Human Sciences of the University of Würzburg.

Note that full information on the approval of the study protocol must also be provided in the manuscript.

Field-specific reporting

Please select the one below that is the best fit for your research. If you are not sure, read the appropriate sections before making your selection.

☐ Life sciences ☒ Behavioural & social sciences ☐ Ecological, evolutionary & environmental sciences

For a reference copy of the document with all sections, see nature.com/documents/nr-reporting-summary-flat.pdf

Behavioural & social sciences study design

All studies must disclose on these points even when the disclosure is negative.

Study description	Quantitative experimental
Research sample	For study 1 we recruited 1,090 Prolific workers, out of which 1050 finished the experiment. The sample of study 1 was not representative. For study 1, participants indicated an average age of 33.0 years (SD = 11.5 years). Self-reported gender identity for study 1: 555 males, 489 females, 5 non-binaries, 1 prefer not to say. The majority of the sample indicated a university degree as their highest level of education (3 no formal qualification, 53 secondary education, 265 high school, 500 bachelor, 195 master, 28 PhD, 6 prefer not to say). Participants reported about 60 different nationalities, with South Africa (n = 262), the UK (n = 174) and Poland (n = 76) mentioned most frequently. For study 2, we recruited 1456 Prolific workers, out of which 1367 finished the experiment and 137 participants had to be excluded because they failed the attention check (final sample size: 1230). We exclusively recruited participants from the UK and our sample was representative for the UK population in terms of age, gender, and ethnicity. For study 2, participants indicated an average age of 47.3 years (SD = 15.6 years). Self-reported gender identity for study 2: 595 males, 619 females, 10 non-binaries, 6 prefer not to say. The majority of the sample indicated a university degree as their highest level of education (12 no formal qualification, 146 secondary education, 325 high school, 532 bachelor, 167 master, 40 PhD, 8 prefer not to say). 385 participants (31.3 %) indicated that they would describe somethelf as a patient. 80 participants (6.5 %) indicated that they work in a healthcare related profession or received a healthcare related training. As our manuscript addresses the public perception of digital medical advice when AI is believed to be involved in advice generation, we recruited members of the general public as our participants.
Sampling strategy	For study 1, convenience sampling was used as our sampling strategy. The study was published on Prolific and all eligible participants could take part until the desired sample size was reached. The sample size for study 1 was based on a formal power analysis (1-β = 95% for d = 0.27, α = .05, two-sample t-test, two-tailed testing, computed in R, version 4.1.1, via the power.t.test function of the stats package). For study 2, we used stratified sampling as our sampling strategy. Therefore, we used the "representative sample" function of Prolific. The intended sample size was stratified across three demographics: age, sex and ethnicity. Based on census data from the UK Office of National Statistics the sample was divided into subgroups with the same proportions as the UK population. Also the sample size of study 2 was based on a formal power analysis (1-β = 90% for d = 0.27, α = .01, two-sample t-test, two-tailed testing, computed in R, version 4.1.1, via the power.t.test function of the stats package).
Data collection	Participants conducted the experiment on their private computer. Participants were blind to the hypotheses and due to the

Data collection	between-subjects setup demand effects should be highly limited. The researchers were not blind to the hypotheses, however there was no direct contact between the researchers and the participants due to the online setup.
Timing	For study 1, all data was collected on the 23rd of January, 2024. For study 2, data was collected between 30rd of April and 2nd of May, 2024.
Data exclusions	For study 1, we only excluded datasets of participants, who did not finish the study (n = 40). For study 2, we excluded datasets of participants who did not finish the study (n = 89) and participants who failed the attention check (n = 137).
Non-participation	For study 1, 3.7 % of participants (n = 40) and for study 2, 6.1 % of participants (n = 89) dropped out. No reasons for dropout were provided but might include technical issues like loss of internet connection or reduced interest in study participation after receiving more detailed information on the experiment.
Randomization	Participants were randomly allocated to the different author label conditions. However, to ensure equally large group sizes participants could not be allocated to a group if all respective slots were already filled.

Reporting for specific materials, systems and methods

We require information from authors about some types of materials, experimental systems and methods used in many studies. Here, indicate whether each material, system or method listed is relevant to your study. If you are not sure if a list item applies to your research, read the appropriate section before selecting a response.

Materials & experimental systems

n/a	Involved in the study
☒	☐ Antibodies
☒	☐ Eukaryotic cell lines
☒	☐ Palaeontology and archaeology
☒	☐ Animals and other organisms
☒	☐ Clinical data
☒	☐ Dual use research of concern
☒	☐ Plants

Methods

n/a	Involved in the study
☒	☐ ChIP-seq
☒	☐ Flow cytometry
☒	☐ MRI-based neuroimaging

Plants

Seed stocks	*Report on the source of all seed stocks or other plant material used. If applicable, state the seed stock centre and catalogue number. If plant specimens were collected from the field, describe the collection location, date and sampling procedures.*
Novel plant genotypes	*Describe the methods by which all novel plant genotypes were produced. This includes those generated by transgenic approaches, gene editing, chemical/radiation-based mutagenesis and hybridization. For transgenic lines, describe the transformation method, the number of independent lines analyzed and the generation upon which experiments were performed. For gene-edited lines, describe the editor used, the endogenous sequence targeted for editing, the targeting guide RNA sequence (if applicable) and how the editor was applied.*
Authentication	*Describe any authentication procedures for each seed stock used or novel genotype generated. Describe any experiments used to assess the effect of a mutation and, where applicable, how potential secondary effects (e.g. second site T-DNA insertions, mosiacism, off-target gene editing) were examined.*
